# Psychological supports for people living with a rare disease in Ireland: an online survey-based study

**DOI:** 10.1007/s11845-025-03902-x

**Published:** 2025-02-26

**Authors:** Darragh Nerney, Emer O’Malley, Caroline Kenny, Alana Ward, Geraldine Sweeney, Vicky McGrath, Laura Egan, Eileen Treacy

**Affiliations:** 1 National Rare Diseases Office, Mater Misericordiae University Hospital, Dublin, Ireland; 2 Mater Misericordiae University Hospital, Dublin, Ireland; 3 Rare Diseases Ireland, Dublin, Ireland; 4 Rare Ireland Family Support Network, Westmeath, Ireland; 5 School of Medicine, Trinity College Dublin, Dublin, Ireland; 6 School of Medicine, University College Dublin, Dublin, Ireland

**Keywords:** Access to psychological services, Genetics, Rare diseases

## Abstract

**Background:**

People living with rare diseases have reported high unmet support needs for access to psychological services despite the significant impact rare diseases have on mental health.

**Aims:**

This study aimed to explore experiences in accessing psychological supports in the Republic of Ireland, and ways in which supports can be improved.

**Methods:**

An online survey was distributed to people living with rare diseases through Irish rare disease patient organisations and expert centres (May–June 2023). Paediatric and adult participants were included with carers providing information on behalf of those age < 18 years. A PRISMA-based scoping review was conducted to explore reported gaps in psychological supports for people living with rare diseases.

**Results:**

Eligible responses were received from 142 participants (87 adults, 55 children, 94 females, 47 males). People living with rare diseases reported a need for psychological supports at all stages of their patient journey. Participants indicated that a rare disease has an impact on educational, social, and financial aspects of daily living. A lack of understanding of the rare disease by healthcare professionals, extended waiting times, and the financial burden of accessing supports were key themes identified by participants.

**Conclusion:**

Living with a rare disease is associated with an increased mental health burden. Gaps remain in the provision of psychological supports for people affected by rare diseases. The integration of mental health supports into the care of people living with rare diseases should be a priority for the Irish health service.

**Supplementary Information:**

The online version contains supplementary material available at 10.1007/s11845-025-03902-x.

## Background

A rare disease is defined in the European Union (EU) as a disease that affects no greater than 5 per 10,000 people [1]. More than 6000 rare diseases have been identified to date collectively affecting approximately 30 million people in Europe and 300,000 people in Ireland, representing a significant health burden [2, 3]. Living with a rare disease can have a significant impact on psychosocial and health-related quality of life [4, 5]. A recent systematic review reported higher prevalence rates of anxiety and depression amongst rare disease patients compared to the general population [6]. In a EURORDIS-Rare Diseases Europe survey, 37% of persons living with a rare disease reported feeling unhappy or depressed often or very often [7]. In addition, those with diagnostic delay (DD) report increased psychological care needs compared to those diagnosed in less than one year [8]. In Ireland, timely diagnosis of rare diseases is a key priority of the Health Care Executive (2019) (HSE) Model of Care for Rare Diseases. However, 37% of rare disease patients in Ireland are reported to spend over 5 years seeking a diagnosis [3, 9].

The ability of people living with rare disease to deal with the many elements of symptoms, treatments, and lifestyle changes requires a patient-centred, integrated, and coordinated approach [10]. However, there is lack of a multidisciplinary coordinated approach to advocating for, and providing psychological support for people living with rare diseases [11–13]. In a previous study, only 12% of people living with a rare disease reported that they used a care coordinator [14]. Unfortunately, most people with rare diseases (RDs) do not receive the support of a professional psychologist, despite the fact that studies suggest people living with a rare disease have an increased need for support [10, 15, 16]. Recent articles from the UK, USA, and Germany reported that RD patients have high unmet support needs when it comes to psychological services [15, 17, 18]. A similar picture is noted in Ireland, with psychological supports often limited to restricted scenarios (e.g., hospital-based) [19]. Psychological services are now provided in each of the 91 Children’s Disability Network Teams (CDNTs) that provide community-based services in the country. However, significant gaps remain for both adult and paediatric RD patients [19].

People living with rare diseases often feel that the psychosocial impact is not noticed or given the weight it deserves by clinicians [11]. One-quarter of patients considered that clinicians had no awareness of the cost involved in psychological assistance. This highlights the clear gap in communication between the patients and healthcare providers in relation to psychological support [12]. There is a distinct need for early, timely, preventative support. Patients have also stated that as the disease progresses, families, and individuals living with rare conditions often feel they have less time to prioritise attending psychological support [16]. In Ireland, patient/service-user representatives identified holistic care that encompasses patients’ and carers’ psychosocial needs as a priority in the development of care pathways for rare diseases [19].

The current study aims to utilise responses from an online survey to explore the landscape of psychological supports for people living with rare diseases in Ireland. This includes information on types of supports, any barriers to access, and ways in which psychological supports may be improved with a view to assisting national rare disease policy.

## Methods

### Scoping review procedure

**Fig. 1:**
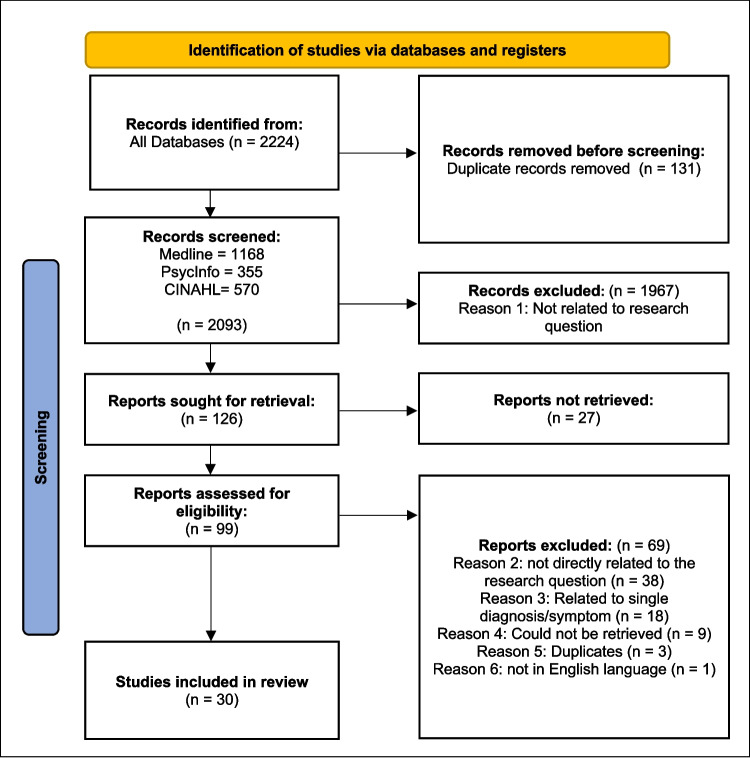
PRISMA (Preferred Reporting Items for Systematic Reviews and Meta-Analyses) flow chart for the search

A scoping review guided by the Preferred Reporting Items for Systematic Reviews and Meta-Analyses extension for Scoping Reviews (PRISMA-ScR) guidelines was conducted to ascertain the full range and nature of studies pertaining to the needs and gaps in psychological supports for people living with rare diseases [20]. PubMed, Psych info, and CINAHL databases were searched in December 2023 and January 2024. Qualitative and quantitative studies in English were eligible. Thirty studies were included in review. All included studies reported on the need for some form of psychological support. Four themes were identified: 1) unmet needs, 2) need for coordinated care, 3) importance of increasing connectedness, and 4) reflections and suggestions for future practise. The PRISMA flow chart for the search is shown in Fig. 1. The list of selected articles is available as Appendix 1 (Supplementary Material).

#### Online survey procedure

An online survey was distributed amongst the Irish RD community with support from National patient organisations representing RD patients (Rare Diseases Ireland—https://rdi.ie/ and Rare Ireland Family Support Network—https://www.rareireland.ie/). The survey was distributed through these organisations’ media channels. Websites and Twitter (X) accounts were used to advertise the study and share the online survey links. Expert centres dedicated to the management of a particular RD or groups of RDs were also contacted to distribute the study advertisement sheet amongst their patients. These expert centres were identified through Orphanet Ireland. A link to the survey was also published on the Orphanet Ireland website (https://orphanet.site/ireland). The online survey was live for 4 weeks from 20 May 2023 to 23 June 2023. The median time taken to complete the survey was 7.4 min (range, 2.5–137.5 min).

Informed consent was obtained from all individuals prior to study participation. Ethical approval for the study was granted by the Mater Misericordiae University Hospital (MMUH) Research Ethics Committee (REC) (reference number: 1/378/2313). A data protection impact assessment was undertaken and approved by the Data Protection Officer at the Mater Misericordiae University Hospital.

The online survey contained 26 single-choice, multiple choice, and open-answer questions. Two versions of the survey were created; one version for people living with RDs and a second for family members/caregivers to answer on behalf of the person living with a RD whom they care for.

The online survey initially included demographic questions to characterise the participants (age, gender, ethnicity, and geographic setting). RD diagnoses were classified initially according to the European Reference Network (ERN) structures. The responses were subsequently classified according to Orphanet medical speciality groupings. Questions on psychological supports in the survey were based around barriers to access, experiences in using supports, and ways in which current supports are effective or can be improved. The online survey questions were reviewed by representatives from RD patient advocacy groups (Rare Diseases Ireland and Rare Ireland Family Support Network) and a senior clinical psychologist to ensure that they were suitable for use.

#### Participants

Participants included people living with an RD and caregivers/family members of people living with an RD. Caregivers responded on behalf of the person living with an RD for whom they care in certain cases, e.g., individuals aged less than 18 years, and individuals who were unable to complete an online survey due to their RD. Inclusion criteria included being aged 18 and over, living with an RD, or caring for someone living with an RD, and being resident in Republic of Ireland. Exclusion criteria included lacking capacity to consent.

A total of 142 complete responses were received to the online survey (87 adults, 55 children, 94 females, 47 males, 1 opted not to declare gender). Responses were received from 81 caregivers and 61 people living with rare diseases. Table 1A summarises the participant characteristics.

**Table 1:** A Characteristics of the study cohort. B Management of mental health disorder(s)

(A)		
	**Accessed PS**^(a)^	**Did not access PS**
	*n*** (%)**	*n*** (%)**
Age		
<18	28 (38.4)	27 (39.1)
18–24	9 (12.3)	5 (7.2)
25–34	7 (9.6)	4 (5.8)
35–44	10 (13.7)	8 (11.6)
45–64	19 (26)	23 (33.3)
>65	0 (0)	2 (2.9)
Gender		
Female	40 (54.8)	54 (78.3)
Male	32 (43.8)	15 (21.7)
Prefer not to say	1 (1.4)	0 (0)
Ethnicity		
Irish	66 (90.4)	60 (87)
Any other white background	5 (6.8)	9 (13)
African	1 (1.4)	0 (0)
Other	1 (1.4)	0 (0)
Geographic setting		
Urban	26 (35.6)	21 (30.4)
Suburban	27 (37)	17 (24.6)
Rural	20 (27.4)	31 (44.9)
(B)		
	**Adult**(>18 y) *n*** (%)**	**Paediatric (<18 y) ** *n***(%)**
Diagnosed with a mental health disorder due to their rare condition		
Yes	24(28.2)	8(14.5)
No	58(68.2)	44(80)
Prefer not to say	3(3.5)	3(5.5)
Prescribed medication for the mental health disorder		
Yes	22(81.5)	3(27.3)
No	5(18.5)	6(54.5)
Prefer not to say	0(0)	2(18.2)
Offered psychological supports in relation to the mental health disorder		
Yes	11(42.3)	6(60)
No	15(57.7)	4(40)
Prefer not to say	-	-

^a^*PS* psychological supports

#### Data analysis

Statistical analysis was performed using the Statistical Package for Social Sciences (SPSS) 27.0 for Windows (SPSS Inc., IBM, New York). Descriptive statistics were performed using frequencies and percentages. Categorical data was analysed using the chi-squared (*χ*^2^) test or, where there was a small sample size, the Fishers’ exact test.

## Results

The characteristics of the 142 persons with RDs analysed are shown in Table 1A below. Psychological supports were attended by 51.4% of participants previously.

**Fig. 2:**
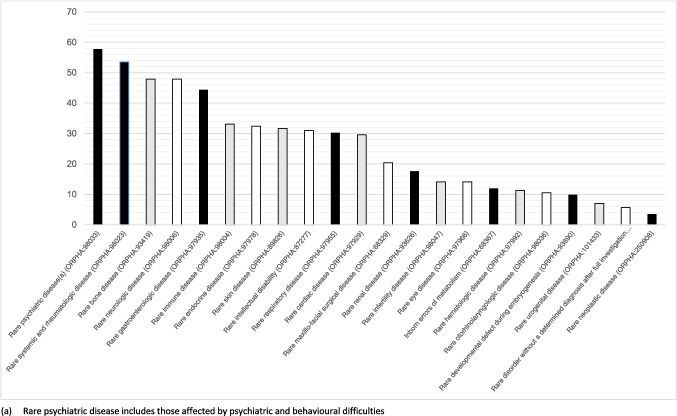
Systems affected according to the Orphanet medical speciality groupings (%)

Participants were asked to indicate which medical system(s) are affected by their RD. The most common system affected was Rheumatology (53.5%), followed by Neurology (47.9%) and Orthopaedics (47.9%). Seventy-six percent (76%) of diagnoses reported by participants are classified as genetic according to Orphanet, the international portal for rare diseases. Participants reported a mean (*M*) of 5.4 medical systems affected (range 1–17) with 57% reporting ≥ 5 systems affected and 9.9% reporting ≥ 10 systems affected. Additional information on systems affected classified according to Orphanet medical speciality groupings is shown in Fig. 2. The majority of individuals still seeking a diagnosis were aged < 18 years (75%), and male (62.5%).

Thirty-two (22.9%) individuals were diagnosed with a mental health disorder as a result of their RD. Of these, 65.8% reported that they were prescribed medication for the disorder and 47.4% reported that they were offered psychological supports (see Table 1B). A higher proportion of adults were prescribed medication for their mental health disorder (81.5%) compared to paediatric individuals (27.3%). Psychological supports were offered to a higher proportion of paediatric individuals (60%) compared to adults (42.3%).

Psychological supports were accessed by 51.4% of individuals (see Table 2A). Psychological supports were needed most post-diagnosis of RD for both adult (40.2%) and paediatric (43.4%) individuals.

**Table 2:** A Access to psychological supports for people with rare diseases. B Barriers to access for psychological supports

(A)		
	**Adult**(>18y) *n***(%)**	**Paediatric (<18y)** *n***(%)**
Accessed psychological supports		
Yes	45(51.7)	28(50.9)
No	42(48.3)	27(49.1)
When psychological supports were needed most		
Post-diagnosis	35(40.2)	23(43.4)
Diagnosis	18(20.7)	11(20.8)
Pre-diagnosis	18(20.7)	10(18.9)
Other^(a)^	7(8)	3(5.7)
Awaiting treatment	9(10.3)	6(11.3)
How psychological supports were found		
Referral	18(40)	19(67.9)
Word-of-mouth	8(17.8)	5(17.9)
Internet search	11(24.4)	1(3.6)
Other^(b)^	8(17.8)	3(10.7)
Referral type		
Self-referral	23(51.1)	6(21.4)
Public referral	14(31.1)	13(46.4)
Private referral	4(8.9)	5(17.9)
Other^(c)^	4(8.9)	4(14.3)
Type of psychological supports accessed		
Counselling services^(d)^	40(43.5)	18(29.5)
Psychology services	26(28.3)	21(34.4)
Social work services	9(9.8)	9(14.8)
Mental health nursing	5(5.4)	5(8.2)
Other^(e)^	12(13)	8(13.1)
How psychological supports were accessed		
Public	22(34.9)	20(57.1)
Private	27(42.9)	9(25.7)
Charitable resource	14(22.2)	4(11.4)
Prefer not to say	0(0)	2(5.7)
Community- or hospital-based supports		
Community-based	35(68.6)	20(69)
Hospital-based	16(31.4)	9(31)
In-person or virtual psychological supports		
In-person	30(69.8)	23(85.2)
Both	11(25.6)	1(3.7)
Virtual	2(4.7)	3(11.1)
Psychological supports were helpful		
Strongly agree	15(34.1)	3(10.7)
Agree	12(27.3)	6(21.4)
Neither agree or disagree	10(22.7)	6(21.4)
Disagree	4(9.1)	6(21.4)
Strongly disagree	3(6.8)	3(10.7)
(B)		
	**Adult** (>18y) *n*** (%)**	**Paediatric (<18y)** *n***(%)**
Attending psychological supports caused a financial burden		
Yes	23(51.1)	8(28.6)
No	21(46.7)	20 (71.4)
Prefer not to say	1(2.2)	0(0)
Waiting time to access psychological supports^(f)^		
0–3 months	30(63.8)	5(16.1)
4–6 months	6(12.8)	3(9.7)
7–12 months	2(4.3)	7(22.6)
13–18 months	2(4.3)	4(12.9)
18 months	7(14.9)	12(38.7)
Time taken to travel to psychological support		
0–60 min	35(92.1)	18(78.3)
61–120 min	2(5.3)	3(13)
120 min	1(2.6)	2(8.7)

^a^Other times included at times of serious stress (e.g., bereavement) and at times of acute illness. Multiple participants indicated that psychological supports will be a future requirement

^b^Other methods included employment assistance programmes and own research

^c^Other referral types included GP referral

^d^Counselling services include professionals offering counselling supports accredited by the Irish Association for Counselling and Psychotherapy (IACP)

^e^Other supports accessed included addiction services, play therapy, art therapy, and psychiatry

^f^9(12.3%) individuals were unsure of their waiting time to access psychological supports

Counselling services was the most common type of psychological support accessed by adult individuals (43.5%). Psychological services were the most common supports accessed by paediatric individuals (34.4%). Adult psychological supports were most commonly accessed privately (42.9%). Paediatric psychological supports were most commonly accessed publicly (57.1%). For those who accessed adult psychological supports, 61.4% found the support(s) to be helpful (“agree” and “strongly agree”). Almost one-third (31.4%) of individuals who accessed paediatric supports found them useful.

Attending psychological supports caused a financial burden for 42.5% of people with RDs and/or their caregivers/family members (see Table 2B), predominantly for those who accessed adult services (51.1%). For those who experienced a financial burden, 93.5% accessed psychological supports privately. The most common waiting time to attend psychological supports was 0–3 months (44.9%). However, 77.1% accessed private or charitable services. For those who waited > 12 months to access supports, 40.5% accessed services publicly and 13.9% accessed supports privately. Over half (51.6%) of individuals waited longer than 12 months to access paediatric psychological supports. One participant (2.8%) who accessed psychological supports privately waited longer than 12 months to attend supports compared to eight participants (19%) who accessed psychological supports publicly. Travel time to attend psychological supports was most commonly 0–60 min for both those accessing adult (92.1%) and paediatric (78.3%) services.

The methods of access and waiting times were also assessed according to psychological support used (see Table 3). Psychology services, social work services, and mental health nursing were most commonly accessed publicly. Counselling services were most commonly accessed privately (41.3%). All psychological supports were most commonly accessed in the community. The longest waiting times were for mental health nursing, with 42.9% of individuals waiting longer than 12 months to attend their appointment. The shortest waiting times were for counselling services, with 70.4% of individuals waiting less than 12 months to attend their appointment.

**Table 3:** Methods of access and waiting times according to psychological support

		Counselling services *n* (%)	Psychology services *n* (%)	Social work services *n* (%)	Mental health nursing *n* (%)
How psychology supports were accessed					
Public		26(34.7)	29(53.7)	17(94.4)	9(90)
Private		31(41.3)	16(29.6)	0(0)	0(0)
Charitable resource		17(22.7)	9(16.7)	1(5.6)	0(0)
Prefer not to say		1(1.3)	0(0)	0(0)	1(10)
Community- or hospital-based supports					
Community-based		43(75.4)	33(76.7)	11(61.1)	6(60)
Hospital-based		14(24.6)	10(23.3)	7(38.9)	4(40)
Waiting time to access psychological supports^(a)^					
0–3 months		29(53.7)	16(41)	6(42.9)	2(28.6)
4–6 months		4(7.4)	3(7.7)	2(14.3)	2(28.6)
7–12 months		5(9.3)	4(10.3)	1(7.1)	0(0)
13–18 months		5(9.3)	2(5.1)	0(0)	2(28.6)
18 months		11(20.4)	14(35.9)	5(35.7)	1(14.3)

^a^9 (12.3%) individuals were unsure of their waiting time to access psychological supports

Five key themes emerged from the open-ended questions included in the survey (see Table 4). Multiple participants identified a lack of understanding of the RD on the part of the healthcare professional (HCP) as a key issue when using psychological supports (Table 4, Q1–Q5). Extended waiting times were identified as a barrier to access by many individuals with one person with an RD waiting five years for an appointment (Q6). Costs were another barrier identified by participants (Q10–Q13). Issues related to costs included prohibitive costs in private sector (Q10), high costs of managing medical aspects of RD (Q11, Q12), and being unable to work due to RD (Q13). Location of psychological supports was another barrier reported by participants (Q13–Q16), particularly for individuals living in rural areas who needed to travel to larger towns/cities to attend psychological supports. Online supports were identified as a useful method of service delivery by multiple participants (Q17–Q20), particularly during the COVID-19 pandemic (Q18). Others identified availability of home-based supports as a key area of improvement for psychological supports in Ireland. One participant described a particular need for home-based supports for elderly RD patients (Q19). Key positive aspects of psychological supports described by participants included having somewhere to express their emotions (Q20, Q21), and learning coping skills to manage their mental health (Q22, Q23).

**Table 4:** Themes and related quotes

Themes	Related quotes
Understanding of rare disease by healthcare professional	Q1.* Participant 45*: “Very narrow viewpoint of the issue at hand. Very little understanding of the impact of the rare condition.”
	Q2.* Participant 53*: “The 2nd psychologist caused my daughter distress due to her misunderstanding of her condition.”
	Q3. *Participant 58*: “My condition is very hidden so can be overlooked and not taken seriously”
	Q4. *Participant 98*: “The nature of the problem isn’t fully understood or appreciated”
	Q5. *Participant 131: *“It took a few counsellors to find the right one that was experienced enough to understand what I was going through.”
Waiting times	Q6. *Participant 29*: “On a waiting list for primary care psychology for approx. 5 years and still has not been seen.”
	Q7. *Participant 55*: “I was on a waiting list for over 2 years and then they wanted me to travel to Dublin for treatment which is not possible
	Q8. *Participant 102*: “Wait times- we need support before it is bad enough to go to A&E.”
	Q9. *Participant 120*: “Waiting times and or the cost of taking time off and travel to Dublin or Galway.”
Costs	Q10. *Participant 2*: “Costs are prohibitive in private sector, waiting lists too long in public…”
	Q11. *Participant 53*: “The cost of the sessions is too much and we just found it hard to manage financially given that we already spend a lot on travel to consultants and medications.”
	Q12. *Participant 73*: “Medical costs are so high that we are left with little left over financially to access care or help.”
	Q13. *Participant 7*: “Carers are left in poverty line, most times we can't work but don't have enough money to pay for services that are extremely expensive.”
Accessibility	Q14. *Participant 58*: “Location due to rural living, wait times still waiting over 18 months on.”
	Q15. *Participant 139*: “Location - living rurally means travelling to larger town/city”
	Q16. Participant 36: “Online counselling more disability friendly.”
	Q17. *Participant 53*: “During COVID lockdowns, the online sessions were helpful.”
	Q18. *Participant 83*: “Online counselling so I didn’t have to travel with pain.”
	Q19. *Participant 115*: “Needs more services coming to homes of elderly—who are not out and about”
Positive aspects	Q20. *Participant 18*: “Having somewhere to express emotions and feelings to someone that could help.”
	Q21. *Participant 45*: “An opportunity to voice difficulties.”
	Q22. *Participant 88*: “Privacy to talk, specific tools to help with my thinking, professional having experience of treating others with my medical condition.”
	Q23. *Participant 113*: “Learning to cope and understand my condition.”

## Discussion

This study aimed to explore the landscape of psychological supports available to people living with rare diseases in Ireland through an online survey. A cohort of both adult (61.3%) and children (38.7%) living with rare diseases participated in the study representing a range of indications (as selected from the European Reference Networks specialities) with input across regional settings in Ireland.

A mental health disorder was diagnosed in 22.9% of participants due to their rare condition (28.2% of adults). This is in line with previous studies that have shown an increased prevalence of affective and anxiety disorders in people living with rare diseases [6]. Participants described a need for psychological supports at all stages of the patient journey, a theme previously identified in Ireland and in other EU countries [8, 16, 19]. The need for psychological support was described at pre-diagnosis, post-diagnosis, and following major treatment for people living with rare conditions. In Ireland, 37% of people living with rare diseases wait over 5 years for a final diagnosis [3]. Diagnostic delay poses a significant burden for people living with rare diseases and their carers. Diagnostic delay was associated with increased anxiety, frustration, and stress amongst an Australian cohort of rare diseases patients [21]. In Spain, a reduction in diagnostic delay was associated with an improvement in psychological impacts such as frustration, irritability, and concentration on daily life in [8]. Improving diagnostic capabilities in Ireland through the introduction of integrated, holistic care pathways, and investment in genomic testing facilities would be of both medical and psychosocial benefit to people living with rare diseases.

Adult psychological supports were most commonly accessed privately (42.9%). Paediatric psychological supports were most commonly accessed publicly (57.1%). Where psychological support was offered at no cost, it was mainly through advocacy groups. Whilst there appears to be significantly reduced waiting times for private access, long waiting times are reported for the public system, especially in relation to paediatric services under the new CDNT system. Overall, 35% of rare disease patients waited > 12 months to access psychological support. For paediatric supports, 52% of paediatric rare disease patients waited longer than > 12 months to access psychological support. A HSE report in Ireland identified 11,000 people on waiting lists for psychological services, with 5000 (45.5%) of those waiting longer than 1 year [22]. The use of private psychological supports amongst 42.9% of adult participants in the current study may be explained by the long waiting times of public care. One participant (2.8%) who accessed psychological supports privately waited longer than 12 months to attend supports compared to 21.4% of participants who accessed psychological supports publicly. Whilst approximately half of the Irish population have private health insurance, 20% has neither private health insurance nor a medical card [23]. High medical fees and other illness-related costs mean that access to private psychological supports is not an option for many rare disease patients and their families. A previous study found that 29% of participants identified being unable to pay for private psychological support that prevented them from accessing professional psychological support [17]. Additionally, a UK report found that those on waiting lists for mental health services experienced exacerbated physical and psychological symptoms [24]. It is imperative that the Irish health service provides adequate resources for psychological services to increase availability and decrease waiting times to access for Irish rare disease patients, optimally coordinated from or linked into rare disease centres of expertise.

Many participants reported their preference for the option of online psychological support, which has increased in availability post COVID-19. People living with rare diseases may be unable to attend in-person supports due to physical disabilities, living in a rural location with inadequate transport links or lack of time due to multiple medical appointments per week. Online communities using platforms such as Facebook and Rare Connect have become a valuable resource to people with rare diseases. In the UK, online support was the most frequently used of all additional sources of support amongst participants in an online survey [17]. Electronic health resources have an emerging key role for people living with rare diseases. Telehealth options and social networking platforms can empower self-advocacy and education [25].

Most participants in the current study sought out psychological supports themselves. Self-referral accounted for 40% of referrals to psychological supports, or through patient advocacy groups. Then, 18% of participants accessed psychological supports via a charitable resource. The high number of referrals via advocacy groups highlights the importance of these organisations in the rare disease population. In Ireland, ‘Rare Diseases Ireland’ and ‘Rare Ireland’ Family Support Network are two such organisations that provide support to people living with rare diseases and their families.

Attending psychological supports caused a financial burden for 42.5% of people with RDs and/or their caregivers/family members. This high figure may be reflected by the number of participants who utilised private psychological supports. A previous study across multiple rare diseases found that catastrophic health expenditure was approximately 36.3% significantly higher than global estimates (12.7%) [26]. Disproportionately higher risk of financial hardship for individuals and families living with rare diseases calls for improved coordination of care and benefits tailored to the rare disease population.

Responses to the open-answer survey questions revealed five major themes: understanding of rare disease by healthcare professional, waiting times, costs, accessibility, and positive aspects of care. These themes align with findings of previous studies which have reported extended waiting times, excessive fees, and lack of awareness of the condition by the healthcare professional as key factors impacting rare diseases’ patients use of supports [4, 17, 27].

## Limitations of the study

More than half of participants in the study were family members/caregivers of a person living with a rare disease answering on their behalf (57%). Increased numbers of people living with rare diseases providing their direct perspectives may have enhanced the study.

It is also noted that the majority of people living with rare diseases included in this study were female (66%) with one-third (33%) of participants identifying as male.

## Conclusions

Living with a rare disease is associated with an increased mental health burden. Psychological supports are needed at all stages of the patient journey. Delays in diagnosis and treatment can negatively impact the lives of people living with rare diseases and their carers. However, gaps remain in the provision of psychological supports for people affected by rare diseases In Ireland. Integrated care which encompasses the physical and psychosocial demands of living with a rare disease is a key priority for the Irish rare disease community with improved access options urgently required.

## Supplementary Information

Below is the link to the electronic supplementary material.

Supplementary file1 (PDF 151 KB)

## Data Availability

The data that support the findings of this study are available in the tables and figures. Further information which might identify individuals due to the nature of the consent is not available. The references for the PRISMA review are available as an Appendix (Supplementary Material), on request.
