# Supplementary material for: Psychological supports for people living with a rare disease in Ireland: an online survey-based study
Source: Ir J Med Sci. 2025 Feb 26;194(2):421–30. doi: 10.1007/s11845-025-03902-x (PMC12031972; doi:10.1007/s11845-025-03902-x)
Supplement: Supplementary file 1 — Supplementary file1 (PDF 151 KB) [file 11845_2025_3902_MOESM1_ESM.pdf]

Appendix 1. Summary of articles selected during PRISMA-Scoping review (n=30)

Author:	Year:	Title of study:	Major themes and Sub themes:	Population:	Number of participants(n):	Country
Anderson M, Elliott EJ, Zurynski YA	2013	Australian families living with rare disease: experiences of diagnosis, health services use and needs for psychosocial support	- Experiences of diagnosis<br>- Health-related function<br>- Use of health services<br>- Impact on family<br>- Support services<br>Information needs	PLWRD	30 families	Australia
Baumbusch J, Mayer S, Sloan-Yip I	2019	Alone in a crowd? Parents of children with rare diseases' experiences of navigating the healthcare system	- The diagnostic journey<br>- Seeking and accessing services<br>Peer support.	Caregivers	16	Canada
Benito-Lozano J, Arias-Merino G, Gómez-Martínez M, Arconada-López B, Ruiz-García B, Posada De la Paz M, Alonso-Ferreira V	2023	Psychosocial impact at the time of a rare disease diagnosis	- Family and marital environment<br>- Physical and mental health needs and care<br>Disease-related personal and social determinants	PLWRD	524	Spain
Boettcher J, Filter B, Denecke J, Hot A, Daubmann A, Zapf A, Wegscheider K, Zeidler J, von der Schulenburg JM, Bullinger M, Rassenhofer M	2020	Evaluation of two family-based intervention programs for children affected by rare disease and their families–research network (CARE-FAM-NET): study protocol for a rater-blinded, randomized, controlled, multicenter trial in a 2x2 factorial design	- Coping level<br>- Relationship level<br>Family dynamics	PLWRD	1236 parents (currently being recruited)	Germany
Boettcher J, Boettcher M, Wiegand-Grefe S, Zapf H	2021	Being the pillar for children with rare diseases — a systematic review on parental quality of life	- Disease-specific predictors of parental QoL<br>- Psychosocial predictors of parental QoL<br>Parental QoL by type of rare disease	Caregivers	N/A	Germany
Bogart K, Hemmesch A, Barnes E, Blissenbach T, Beisang A, Engel P	2022	Healthcare access, satisfaction, and health-related quality of life among children and adults with rare diseases. Orphanet journal of rare diseases	- Rare disease diagnosis and health<br>- Health insurance<br>- Knowledge and support in relation to rare disease<br>- Patient satisfaction<br>Stigma	PLWRD	1128	USA
Crellin E, Martyn M, McClaren B, Gaff C	2023	What matters to parents? A scoping review of parents' service experiences and needs regarding genetic testing for rare diseases	- Clear information, communication, and support for managing child's ongoing care<br>- Partnering with and providing support for families,<br>- Environmental context<br>- Timeliness<br>- Smooth transitions and continuity of care,<br>- Effective care by trusted health providers<br>Emotional support, empathy and respect	Caregivers	N/A	Australia
Currie G, Szabo J	2020	Social isolation and exclusion: the parents' experience of caring for children with rare neurodevelopmental disorders	- Difference and vulnerability<br>- Social taboo and stigma<br>- Systemic disease<br>Incomprehension	Caregivers	15	Canada
Bosi G, De Santis M, Hervás C, Weinman A, Bottarelli V	2019	Patient empowerment of people living with rare diseases. Its contribution to sustainable and resilient healthcare system	- Defining an empowered patient<br>- Health literacy, education and capacity-building<br>- Shared decision-making and Integration of patients' views<br>Self-care and self-management views	PLWRD	N/A	Italy/EU
Gimenez-Lozano C, Páramo-Rodríguez L, Cavero-Carbonell C, Corpas-Burgos F, López-Maside A, Guardiola-Vilarroig S, Zurriaga O	2022	Rare Diseases: Needs and Impact for Patients and Families: A Cross-Sectional Study in the Valencian Region, Spain	- Socio-economic situation of families with rare diseases<br>- Diagnostic and clinical features<br>- Treatment<br>- Expenditures and unmet needs arising from rare diseases<br>Impact on the emotional and social sphere	PLWRD	163	Spain
Llubes-Arria L, Sanroma-Ortiz M, Torne-Ruiz A, Carillo-Alvarez E, Garcia-Exposito J, Roca J	2022	Emotional experience of the diagnostic process of a rare disease and the	- Experiences and perceptions of patients and families<br>External factors: Resources and support systems	PLWRD	N/A	Spain/

		perception of support systems: A scoping review				
Manalel JA, Sumrall S, Davidson H, Grewal M, Granovetter MA, Koehly LM	2024	Stress, coping, and positive aspects of caregiving among caregivers of children with rare disease	- Primary caregiving stressors<br>Coping strategies	Caregivers	214	USA
McConkie-Rosell A, Hooper SR, Pena LD, Schoch K, Spillmann RC, Jiang YH, Cope H, Undiagnosed Diseases Network, Palmer C, Shashi V	2018	Psychosocial profiles of parents of children with undiagnosed diseases: Managing well or just managing?	- Parental inner emotional state<br>- Coping self-efficacy<br>- Health care empowerment<br>Gender differences	Caregivers	50	USA
Mcmullan J, Lohfeld L, McKnight AJ	2022	Needs of informal caregivers of people with a rare disease: a rapid review of the literature	- Caregiver burden<br>- Support through the diagnosis process<br>- Social needs<br>- Financial needs<br>- Psychological needs<br>- Information and communication needs<br>Acknowledgement from healthcare professionals	Caregivers	N/A	Northern Ireland
McMullan J, Crowe AL, Downes K, McAneney H, McKnight AJ	2022	Carer reported experiences: Supporting someone with a rare disease	- Training for carers<br>- Understanding of genetics and multi-omics<br>- Mental health<br>- Medical appointments and HCP<br>Networking, social isolation	Caregivers	N/A	Northern Ireland
Mund M, Uhlenbusch N, Rillig F, Weiler-Normann C, Herget T, Kubisch C, Löwe B, Schramm C	2023	Psychological distress of adult patients consulting a center for rare and undiagnosed diseases: a cross-sectional study	- Psychological distress<br>- Overlap between a positive screening for depression, anxiety and somatic symptom disorder<br>Aspects associated with a positive screening for depression, anxiety, and somatic symptom disorder	Caregivers	167	Germany
Pelentsov LJ, Fielder AL, Laws TA, Esterman AJ	2016	The supportive care needs of parents with a child with a rare disease: results of an online survey.	- Equity in care<br>- Practical care needs<br>- Relationships<br>- Emotions<br>- Social support<br>Financial burden	Caregivers	301	Australia
Rice DB, Carboni-Jimenez A, Canedo-Ayala M, Turner KA, Chiovitti M, Levis AW, Thombs BD	2020	Perceived Benefits and Facilitators and Barriers to Providing Psychosocial Interventions for Informal Caregivers of People with Rare Diseases: A Scoping Review	- Behavioural/psychological interventions<br>- Support-focused interventions<br>- Educational interventions<br>Multicomponent interventions	Caregivers	N/A	Canada/Global
Rihm L, Dreier M, Rezvani F, Wiegand-Grefe S, Dirmaier J	2022	The psychosocial situation of families caring for children with rare diseases during the COVID-19 pandemic: results of a cross-sectional online survey	- Distress of caregivers<br>- Psychosocial information needs of caregivers<br>- Health-related QoL of children with rare diseases<br>Practical implications	Caregivers	149	Germany
Sandilands K, Williams A, Rylands AJ	2022	Carer burden in rare inherited diseases: a literature review and conceptual model	- Living with rare inherited disease<br>- Carer coping strategies<br>Carer needs/burden	Caregivers	N/A	UK
Sestini S, Paneghetti L, Lampe C, Betti G, Bond S, Bellettato CM, Maurizio S	2021	Social and medical needs of rare metabolic patients: results from a MetabERN survey	- Schooling and intellectual disability<br>- Psychological support<br>- Social worker services<br>- Civil disability and economic support<br>- Job opportunities<br>- Transition from childhood to adult care<br>- Specialised medical assistance<br>Medical expenses	PLWRD	924	Italy/Europe
Simpson A, Bloom L, Fulop NJ, Hudson E, Leeson-Beevers K, Morris S, Ramsay AI, Sutcliffe AG, Walton H, Hunter A	2021	How are patients with rare diseases and their carers in the UK impacted by the way care is coordinated? An exploratory qualitative interview study	- Delays/barriers to accessing care<br>- Time and burden<br>- Communication between stakeholders<br>- Patients and carers as coordinators of own care	Caregivers	15	UK

			Physical, financial, and psychosocial impact of uncoordinated care			
Smits RM, Vissers E, Te Pas R, Roebbers N, Feitz WFJ, van Rooij IALM, de Blaauw I, Verhaak CM	2022	Common needs in uncommon conditions: a qualitative study to explore the need for care in pediatric patients with rare diseases	- Family focused care<br>- Coping with uncertainty<br>- Empathic communication<br>- Practical support, information<br>- Psychological support<br>- Social support<br>Interdisciplinary care	PLWRD	12	Netherlands
Somanadhan S, O'Donnell R, Bracken S, McNulty S, Sweeney A, O'Toole D, Rogers Y, Flynn C, Awan A, Baker M, O'Neill A	2023	Children and young people's experiences of living with rare diseases: An integrative review	- Experiences of stigmatization<br>- Self-consciousness<br>- Restrictions in independent living, resilience/coping strategies<br>- Psychological and emotional impact<br>- Social impact versus social connectedness<br>Transitioning to adult healthcare	PLWRD	N/A	Ireland
von der Lippe C, Neteland I, Feragen KB	2022	Children with a rare congenital genetic disorder: a systematic review of parent experiences	- Lack of coordinated care<br>- Responsibilities and challenges<br>- Society's lack of information and knowledge<br>- Changes to everyday life<br>- Parents as coordinators, advocates, and experts<br>- Factors promoting positive experiences in parents<br>- Engaged and understanding HCP<br>- Benefits of social support<br>Protective factors and coping mechanisms	Caregivers	N/A	Norway
Ward AJ, Murphy D, Marron R, McGrath V, Bolz-Johnson M, Cullen W, Daly A, Hardiman O, Lawlor A, Lynch SA, MacLachlan M	2022	Designing rare disease care pathways in the Republic of Ireland: a co-operative model	- Diagnosis<br>- Care<br>Information resources	PLWRD/Caregivers/HCP	N/A	Ireland
Witt S, Schuett K, Wiegand-Grefe S, Boettcher J, Quitmann J	2023	Living with a rare disease - experiences and needs in pediatric patients and their parents.	- Daily life with a rare disease<br>- Experience in the healthcare system<br>- Psychosocial support<br>Difficulties, barriers and improvements	PLWRD	74	Germany
Witt S, Kristensen K, Wiegand-Grefe S, Boettcher J, Bloemeke J, Wingartz C, Bullinger M, Quitmann J, and the CARE-FAM-NET study group.	2021	Rare pediatric diseases and pathways to psychosocial care: a qualitative interview study with professional experts working with affected families in Germany	- Everyday life of children with rare diseases and their families<br>- Known pathways to psychosocial care<br>- Assessment of the adequacy of and access to psychosocial care and the referral practices of professionals<br>- Psychosocial care recipients and situations in which psychosocial care is required and<br>Barriers and suggestions for improvement of psychosocial care	HCP	50	Germany
Witt S, Schuett K, Wiegand-Grefe S, Boettcher J, Quitmann J	2023	Living with a rare disease - experiences and needs in pediatric patients and their parents	- Daily life with a rare disease<br>- Experiences with the health care system<br>- Psychosocial support<br>- Difficulties and barriers<br>Improvements for patient-oriented support	PLWRD/Caregivers	15/74	Germany
Zurynski Y, Deverell M, Dalkeith T, Johnson S, Christodoulou J, Leonard H, Elliott EJ	2017	Australian children living with rare diseases: experiences of diagnosis and perceived consequences of diagnostic delays	- Diagnostic delays and consequences for families<br>- Levels of satisfaction with the way diagnosis was given and psychological support<br>Children without a diagnosis	PLWRD	462	Australia

PLWRD = Persons Living with a Rare Disease; QoL = Quality of Life; HCP = Healthcare Professionals
